# Apical prolapse correction by unilateral pectineal suspension

**DOI:** 10.1007/s00404-023-07067-9

**Published:** 2023-05-26

**Authors:** Michael Schreibmayer, Dimitrios I. Bolovis, Cosima V. M. Brucker

**Affiliations:** 1 University Women’s Hospital, Paracelsus Medical University, Nuremberg, Germany; 2 Department of Obstetrics and Gynecology, Krankenhaus Barmherzige Brüder St.Veit/Glan, St. Veit an der Glan, Austria; 3 Paracelsus Medical University, Salzburg, Austria; 4 Georg Simon Ohm Technical University, Nuremberg, Germany

**Keywords:** UPS, Pectopexy, Unilateral pectineal suspension

## Presentation

Throughout the last decades, surgical treatment of pelvic organ prolapse (POP) has been achieved by a broad variety of methods and modifications. The ongoing debate questioning the use of mesh material and its ban in several countries has encouraged research into mesh-free alternatives [1, 2]. Furthermore, preserving the uterus in the absence of uterine pathology has become an appropriate option [3]. Recently, the method of unilateral pectineal suspension (UPS) was established for uterus-preserving mesh-free apical POP correction [4]. A retrospective analysis of 47 patients revealed excellent short-term results with a subjective and objective treatment success of 93.6% as defined by a standardized composite endpoint [5].

In the image, the key steps of UPS applied to a patient with stage 2 apical prolapse as defined by the pelvic organ prolapse quantification system described by ICS/IUGA [6] are shown. The key steps are exposure of the pectineal ligament and the anterior cervix, followed by placement of a strong non-absorbable suture between the two structures to achieve a physiologic suspension of the uterus.

The anatomical landmarks for the exposure of the pectineal ligament are the obliterated umbilical artery and the round ligament. The pectineal ligament is easily exposed after peritoneal incision at the triangle between the two structures and the pelvic wall (a). The combination of an adjustable slip knot and the transvaginal manipulation of the cervix offers optimal placement of the uterus in its physiological position respecting the vaginal axis (e).

## Discussion

UPS is a fully standardized and easily reproducible method to correct apical prolapse. Therefore, it is suitable for broad implementation as well as training of future pelvic floor surgeons. It can be performed in a time-efficient and, thus, also cost-efficient way, requiring less than 1 h to complete the entire procedure. Due to its minimally invasive nature, it is also a good candidate for day case surgery. The method compares favorably to other methods of POP repair, fulfilling a large panel of quality criteria for POP correction. 
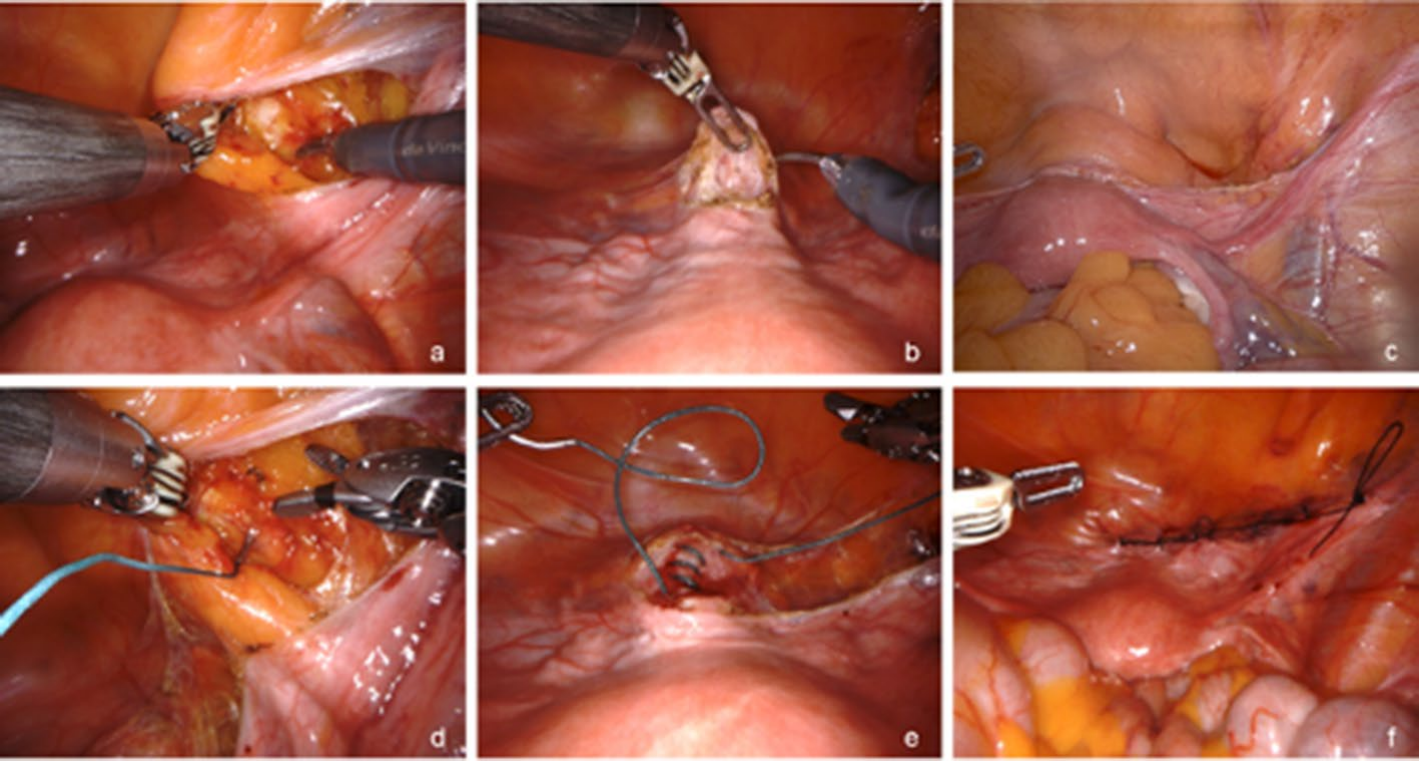


## Image footnote

Exposure of the pectineal (“Cooper”) ligament (a), removing the bladder flap from the anterior cervix (b), completing peritoneal dissection between bladder flap and pectineal ligament (c), placing a non-absorbable Ethibond #2 suture through the pectineal ligament (d), attaching the Ethibond #2 suture to the cervix and connecting the two structures (e), closure of the peritoneum with a running absorbable Vicryl #2.0 suture (f).

## Data Availability

The pictures which are shown were taken during the surgeries in our hospital.
