# Correction: Apical prolapse correction by unilateral pectineal suspension

**DOI:** 10.1007/s00404-023-07262-8

**Published:** 2023-11-03

**Authors:** Michael Schreibmayer, Dimitrios I. Bolovis, Cosima V. M. Brucker

**Affiliations:** 1 University Women’s Hospital, Paracelsus Medical University, Nuremberg, Germany; 2 Department of Obstetrics and Gynecology, Krankenhaus Barmherzige Brüder St.Veit/Glan, St. Veit an der Glan, Austria; 3 Paracelsus Medical University, Salzburg, Austria; 4 Georg Simon Ohm Technical University, Nuremberg, Germany

**Correction: Archives of Gynecology and Obstetrics** 10.1007/s00404-023-07067-9

Figure 1 was missing from this article; the figure should have appeared as shown below. The Original article is also updated.

**Fig. 1:**
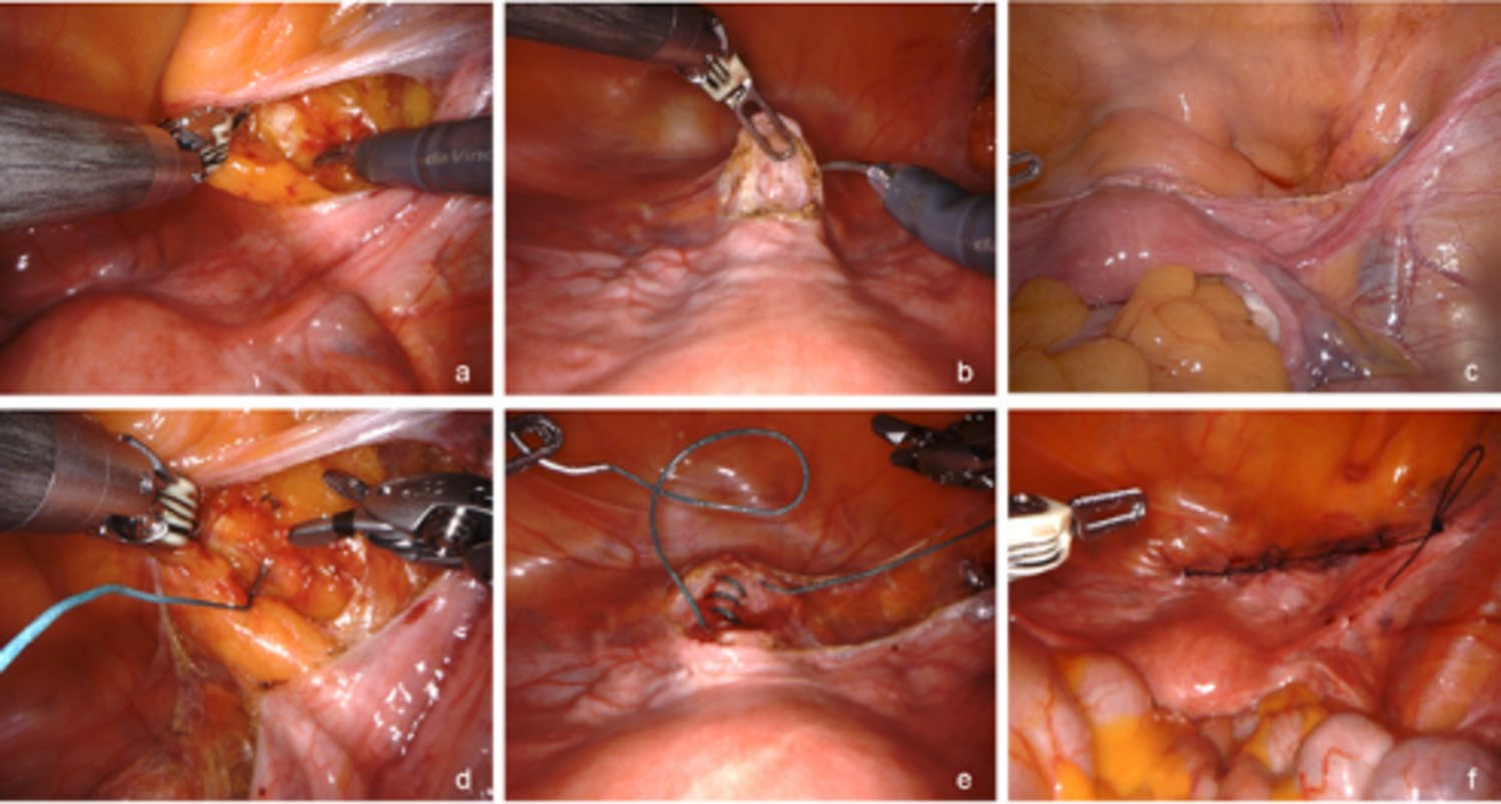
Exposure of the pectineal (“Cooper”) ligament (**a**), removing the bladder flap from the anterior cervix (**b**), completing peritoneal dissection between bladder flap and pectineal ligament (**c**), placing a non-absorbable Ethibond #2 suture through the pectineal ligament (**d**), attaching the Ethibond #2 suture to the cervix and connecting the two structures (**e**), closure of the peritoneum with a running absorbable Vicryl #2.0 suture (**f**)

